# High-Density Genetic Linkage Mapping in Turbot (*Scophthalmus maximus* L.) Based on SNP Markers and Major Sex- and Growth-Related Regions Detection

**DOI:** 10.1371/journal.pone.0120410

**Published:** 2015-03-16

**Authors:** Weiji Wang, Yulong Hu, Yu Ma, Liyong Xu, Jiantao Guan, Jie Kong

**Affiliations:** 1 Key Laboratory of Sustainable Development of Marine Fisheries, Ministry of Agriculture, Yellow Sea Fisheries Research Institute, Chinese Academy of Fishery Sciences, Qingdao, Shandong Province, P. R. China; 2 Ocean University of China, Qingdao, Shandong Province, P. R. China; The Ohio State University, UNITED STATES

## Abstract

This paper describes the development of a high density consensus genetic linkage map of a turbot (*Scophthalmus maximus* L.) family composed of 149 mapping individuals using Single Nucleotide Polymorphisms (SNP) developed using the restriction-site associated DNA (RAD) sequencing technique with the restriction enzyme, PstI. A total of 6,647 SNPs were assigned to 22 linkage groups, which is equal to the number of chromosome pairs in turbot. For the first time, the average marker interval reached 0.3958 cM, which is equal to approximately 0.1203 Mb of the turbot genome. The observed 99.34% genome coverage indicates that the linkage map was genome-wide. A total of 220 Quantitative Traits Locus (QTLs) associated with two body length traits, two body weight traits in different growth periods and sex determination were detected with an LOD > 5.0 in 12 linkage groups (LGs), which explained the corresponding phenotypic variance (*R^2^*), ranging from 14.4–100%. Among them, 175 overlapped with linked SNPs, and the remaining 45 were located in regions between contiguous SNPs. According to the QTLs related to growth trait distribution and the changing of LGs during different growth periods, the growth traits are likely controlled by multi-SNPs distributed on several LGs; the effect of these SNPs changed during different growth periods. Most sex-related QTLs were detected at LG 21 with a linkage span of 70.882 cM. Additionally, a small number of QTLs with high feasibility and a narrow *R^2^* distribution were also observed on LG7 and LG14, suggesting that multi LGs or chromosomes might be involved in sex determination. High homology was recorded between LG21 in *Cynoglossus semilaevis* and turbot. This high-saturated turbot RAD-Seq linkage map is undoubtedly a promising platform for marker assisted selection (MAS) and flatfish genomics research.

## Introduction

Key technological breakthroughs, such as artificial breeding, have allowed for the rapid development of turbot (*Scophthalmus maximus* L.) farming since its introduction into China in 1992. Today, turbot farming is one of the most promising industries in North China’s sea. The estimated global annual production of turbot was 77,117 tons in 2012 (http://www.fao.org/fishery/statistics/software/fishstatj/en), approximately 60,000 tons were produced in China in 2010 [1]. As an introduced fish species, turbot farming in China depends on spawner fish and seedlings at the initiation phase. As a result, selective breeding and genetic improvement have been particularly important in China compared with countries with native turbot populations. However, the Chinese turbot industry did not pay much attention to this issue until recently, when unfavorable traits were observed, such as longer growth period, lower seedling production and disease susceptibility [2–3]. This undoubtedly caused heavy economic losses and hindered the sustainable turbot farming industry that the government has long encouraged. Currently, researchers and farmers agree that genetically improved turbot that display fast growth and/or disease resistance characteristics are crucial for a sustainable and environmentally friendly turbot industry. In 2006, following the success of genetic improvement in European livestock, researchers from the Yellow Sea Fisheries Research Institute (YSFRI) in China, introduced a polygenic genetic evaluation system based on BLUP (Best Linear Unbiased Prediction) to develop genetically improved turbot broodstock lines [4]. Since the introduction of this genetic evaluation system, turbot farming has seen obvious genetic improvement (faster growth). Nevertheless, this genetic evaluation system is more suitable and powerful for traits with medium to high level heritability, such as growth than those with lower heritability, such as disease resistance in aquatic organisms [5–7]. Moreover, because of turbot’s the long sexual maturation phase, it takes approximately 3–4 years to select one generation by this method. Considering these issues, an increasing number of researchers have employed marker-assisted selection (MAS) techniques’ for selective breeding, especially when the target trait has low-level heritability.

As an essential tool in locating growth, sex control, and disease resistance-related genomic regions, genetic linkage mapping is the backbone to MAS program development in aquaculture [8]. The first turbot genetic linkage map consisted of 242 anonymous microsatellites distributed on 26 linkage groups (LG) [9]. Among these, the sex determination, growth rate, and pathogen resistance related QTL regions were developed or identified in subsequent studies [10–13]. In 2010, Ruan constructed another linkage map using anonymous turbot microsatellites and they noted 158 newly developed SSRs in 21 and 30 genetic linkage groups in the male and female maps, respectively [14]. Recently, new, gene-enriched, high-resolution turbot maps were reported. Some of the mapped markers (180) were EST-linked, making this map useful for QTL mapping and comparative genomics research; however, the low average inter-marker distance (3.7 cM; ~2 Mb) might limit its application for MAS projects [8].

The arrival of next-generation sequencing (NGS) techniques, genome-wide molecular marker detection, such as SNPs, has made it possible to construct higher density genetic linkage maps, even in non-model organisms such as the Atlantic salmon (*Salmo salar*): a recent study using the restriction-site associated DNA sequencing (RAD-Seq) technique mapped approximately 6, 000 SNP markers in Atlantic salmon linkage maps [15]. Because of the high throughput nature of SNP detection, RAD has not only been applied to genetic linkage mapping to clarify the sex mechanism of aquatic animals [16], but also to infer phylogenetic relationships among African lake fish species with extensive genetic diversity [17].

In this study, we constructed the first high density SNP linkage map of the turbot genome using SNP markers derived using the RAD-Seq technique. Additionally, growth data, including individual body length and weight, body thickness on the last day, and sex were recorded in four different growth periods, and the sex of the fish were also recorded as well, for QTL mapping analysis. This high-density turbot linkage map will be useful in the following studies: physical map construction in turbot, identifying candidate genes associated with growth traits, clarifying the mechanism of sex-determination and MAS for selective breeding in turbot farming.

## Materials and Methods

### Ethics Statement

This research was approved by the Animal Care and Use committee in the Yellow Sea Fisheries Research Institute, Chinese Academy of Fishery Sciences; all of mapping family individuals, including the parents were anesthetized in sea-water with 100 mg/L Tricaine for 12 min before fin clipping, then all fish were dissected for gonad anatomy, through which, the individuals’ sex were determined. At last, all fish were stored at -20°C until required. All *in vivo* experiments in present study were in accordance with the Animal Research: Reporting *In Vivo* Experiments (ARRIVE) guidelines. The animals used in this study were turbot (*Scophthalmus maximus* L.), that originated from European countries, including Spain, France, and Norway; the species was introduced into China for farming around 1992 and is not an endangered or protected fish species both in either China or its native countries. All of the scientific activities in this study were in accordance with the Law of the People’s Republic of China on the Protection of Wildlife (http://www.china.com.cn/chinese).

### Mapping family and data collection

The mapping family used in this study was established on May 9, 2012 via artificial insemination in a Yellow Sea Fishery Company (Haiyang city, Shandong Province, P. R. China) hatchery plant, where a selective turbot-breeding project was initiated in 2006 using introduced stocks. The parents were fin-clipped and stored at -75°C for genomic DNA isolation. All of the filial fry (approximately 1, 500) were cultured in a fiberglass-reinforced plastic tank containing 500 L natural sea water until 100 days post-fertilization, at which point they were tagged with visible implant elastomer (VIE) (http://www.nmt.us/) for individual identification and transferred to a 3 m^3^ cement tank. During the entire culture period, growth data (body length, body weight) was recorded for each individual on the following dates: Dec. 14, 2012; Feb. 26, 2013; May 24, 2013; and July 2, 2013. On the last day, 149 individuals were randomly selected as the mapping family, and the body thickness of these individuals was also recorded. The mapping individuals were fin-clipped for DNA extraction, and the gonad was used to identify their sex. During the fin clipping and sex determination processes, all of the fish were in anesthetized before they were euthanized.

### RAD library construction and Illumina HiSeq2000 sequencing

Genomic DNA was isolated according to Wang [18], with minor adjustments. Genomic DNA samples from each individual were quantified and measured using GeneQuant (Amersham Biosciences Ltd.) (http://www.gelifesciences.com) and 0.7% agarose electrophoresis. A total of 151 RAD libraries were prepared, two parent libraries and 149 filial libraries. RAD libraries were prepared according to Etter [19]. The restriction enzyme, Pst I, was used to digest the genomic DNA. Briefly, within each library, the first (P1) adaptor containing an individual-specific nucleotide barcode was ligated to the genomic DNA of each sample. The samples within each library were then pooled and the pools were sheared to ~ 400 bp fragments using a sonicator, then 250~500 bp fragments were then selected by agarose electrophoresis. An adapter (P2) with divergent ends was used to ligate to the fragments, and the libraries were subjected to an 18-cycle PCR amplification. Finally, 300 bp to 500 bp fragments were selected. A total of 152 multiplexed sequencing libraries were constructed, and each DNA sample was assigned a unique nucleotide multiplex identifier (MID) for SNP barcoding. Single-end (101 bp) sequencing was performed using the Illumina HiSeq2000 (http://www.illumina.com/) in a total throughput of 152 lanes. For Illumina pair-end sequencing of each strain, at least 3 μg of genomic DNA was used to construct the sequencing libraries. Paired-end libraries with inserts of ~300 bp were prepared following Illumina’s standard genomic DNA library preparation procedure. Purified genomic DNA was sheared into smaller fragments with Covaris, and blunt ends were generated with T4 DNA polymerase. After adding an ‘A’ base to the 3′ end of the blunt phosphorylated DNA fragments, adapters were ligated to the ends of the DNA fragments. The desired fragments were purified by gel-electrophoresis, followed by selective enrichment and PCR amplification. The index tag was introduced into the adapter at the PCR stage, and we performed a library quality test. Finally, the qualified Illumina pair-end library was used for Illumina Hiseq2000 sequencing (100*2).

Raw sequence reads were trimmed to 85 nucleotides from the 3’ end, ensuring that more than 97.5% of the nucleotides had a quality value above Q30 (0.1% sequencing error). The trimmed reads were clustered into read tags (RAD-tags) by sequence similarity using *ustacks* to produce unique candidate alleles for each RAD locus [20]. A maximum base-pair mismatch of one was allowed in this step for the genetic mapping population. RAD-tags were then collapsed into clusters using *ustacks* under default parameters for SNP calling.

### SNP discovery, filtering, genotyping and validation

Each raw sequencing data (101 bp) was end-sheared to an 85 bp sequenced fragment. For higher confidence SNP genotyping results, the following parameters were settled during the RAD-tag comparison within and among individuals: the minimum stack depth was at least 30, a maximum of two mismatches were allowed in an individual locus and one mismatch between alleles. Raw sequences are available from the Sequence Read Archive (SRA, http://www.ncbi.nlm.nih.gov/sra/), and the accession number is SRP050882.

### Linkage map construction and QTL mapping

Genotype calling refers to the process of determining the SNP loci genotypes of each individual after SNP calling, as described by Hohenlohe [21]. Customized perl scripts were applied to generate a “.loc” format file, which served as the input file for JoinMap 4.0 [22]. Markers with > 66% successful calls (50 missing data at most) were collected for further segregation analysis. Three types of SNP genotyping were used in the genetic linkage mapping: SNPs are heterozygous in maternity (*lm*) and homozygous in paternity (*ll*), progeny segregation was recorded for maternal linkage mapping; similarly, SNPs that were homozygous in maternity (*nn*) and heterozygous in paternity (*np*) were recorded for paternity linkage mapping; and SNPs that were heterozygous in both maternity and paternity (*hk*) were recorded as anchor markers for the final consensus linkage map. Only segregation ratios of 1:1 in the first two types and 1:2:1 in the third type (*p* > 0.05) were used for linkage mapping. The LOD threshold value for segregated SNP marker grouping was 6.0. During the linkage analysis, a fast Monte Carlo multipoint maximum likelihood algorithm for crosses between inbred lines was adopted [23], which was then extended suitable for a full-sib family and built into the JoinMap4.0 [22–23]. Considering that a large number of segregated SNP loci were involved in the present linkage analysis, map order optimization uses a general Monte Carlo optimization method called simulated annealing to minimize the sum of recombination frequency (RF) in adjacent segments, using the sum of RFs rather than the likelihood enormously reduces the computations [24, 25]. SNPs with segregation types *lm*×*ll* and *nn*×*np* provide complete information of maternity and paternity, and were used in paternal and maternal linkage map construction. The anchor markers (*hk*×*hk*) “bridge” paternal and maternal maps creating an integrated map, on which, map distances between anchor markers were taken as the average of the two parental distances; Paternal and maternal map markers were placed on integrated map by either interpolation (markers positioned between anchor markers) or extrapolation (markers positioned distal to the outermost anchor markers), they maintain the distances to the anchor markers as they were in the parental maps [23]. All of the above, including RF estimation, map order optimization, and parental map integration etc., were completed in JoinMap4.0 [22]. Map distance was calculated in centiMorgans (cM) using the Kosambi mapping function. Four growth measurements of body length (L1–L4) and body weight (W1–W4) at different ages, body thickness (T) of each individual on the fourth sampling date, and sex data were constructed into a data matrix, which was applied for QTL mapping using MapQTL 5.0 [26]. QTL region detection, the percentage of the phenotypic variance explained, and the genotypic information coefficient (*GIC*) were calculated with the interval QTL mapping model (IM) [27]. In the QTL mapping step, the LOD threshold for testing the significance of the QTL peaks were calculated using 1, 000 permutations for each of the trait data sets and a genome-wide significant level of 5%. For interval distances > 1.0 cM, significant thresholds were estimated every 1.0 cM. A default threshold of odds (LOD) value of 3.0 and above indicated a QTL locus in the present study. Considering that the present QTL mapping was based on a single mapping family, these QTLs should also be tested for significance in other mapping families or populations, QTLs with an LOD > = 5.0 were used in the present study. However, QTLs with LOD from 3.0 to 5.0 were not discarded; they were just assigned to other trials.

## Results

### RAD sequencing and genotyping

The mapping family parents were unrelated individuals from two stocks from Denmark and France consisting of 149 individuals. To increase the chances of detecting more segregated SNPs, the parents were sequenced with a higher depth (14.77 × and 11.55 ×) than the offspring (mean = 8.00 ×). The average read length was approximately 95 in both the offspring and the parents; the average raw read number was approximately 5, 13, and 9 million for the offspring, female parent, and male parent, respectively. The RAD-tag number was approximately 0.6–0.7 million per individual for both offspring and parents (Table 1). A missed genotype number above 50 on any SNP was filtered. In total, 13, 697 SNP loci were located, among which, 11, 289 SNPs were identified with no missing genotype data, and 1, 313 SNPs were identified with one missing genotype.

**Table 1:** *S*. *maximus* RAD sequencing results.

	Read Length (bp)	Raw Length data (bp)	Raw Reads Number	Rad-tag Number	Average depth
**Mapping filial offspring (Average value)**	94.78	538,046,787	5,675,812	622,045	8.00
**Maternity**	95	1,250,602,040	13,164,232	743,630	14.77
**Paternity**	95	916,746,390	9,649,962	694,637	11.55

### Linkage map construction

**Fig 1:**
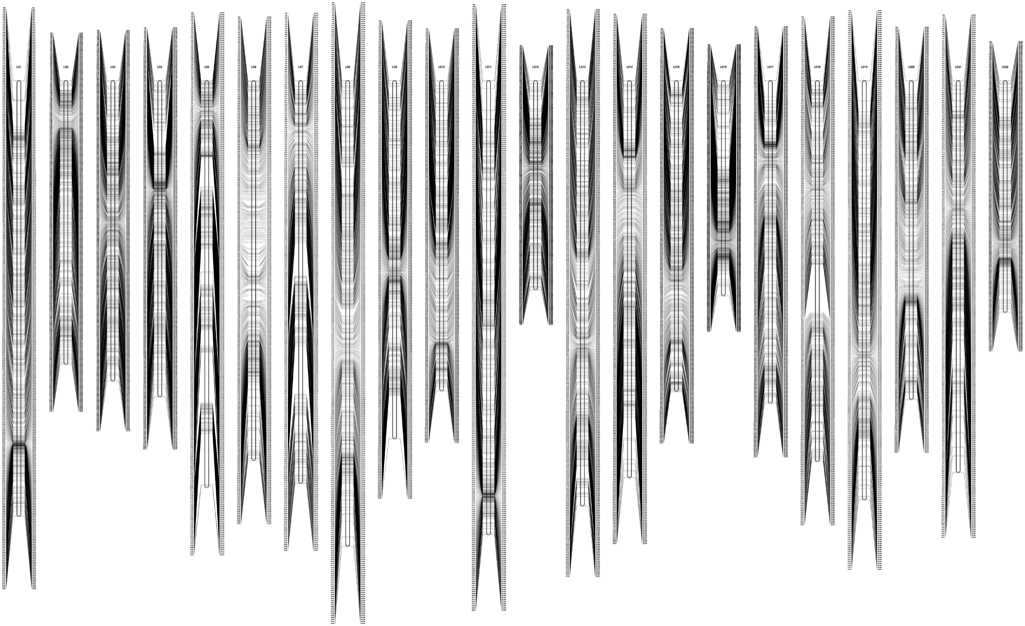
High-density genetic linkage map of turbot (*S*. *maximus*) SNPs. Linkage group name and number are placed above each linkage group, for example, LG2 represents the second linkage group (LG). Numbers on the left are recombination fraction (RF) between the uppermost SNP mapped in an LG to any linked SNP marker in Kosambi’s distance in the same LG. For example, the left third number 2.796cM in LG3, represents the genetic distance between the uppermost SNP marker SM_670 and the third SNP marker SM_672. Linked SNP’ names are on the right, SM *Scophthalmus maximus*, and the number is the order of SNPs 1 to 6, 647.

Using the retained 13, 697 SNP loci, different types of loci were assigned to construct maternal and paternal linkage maps. A heterozygous SNP from both maternity and paternity was used as a “bridge” to construct a consensus map. The final consensus linkage map consisted of 4, 667 SNPs from maternity, 1, 041 SNPs from paternity and 939 heterozygous SNPs were common to both maternity and paternity. The average number of missed genotype information in the three SNP segregation matrices was 18, 23, and 18, respectively (Table 2). In the final turbot consensus linkage map, 6, 647 SNPs were assigned to 22 linkage groups, which is equal to the number of chromosome pairs in turbot (Fig. 1). This map is 2622.09 cM (*G*_*oa*_) in length, with an average marker interval of 0.3958 cM, which is equal to 0.1203 Mb of the turbot genome, the whole genome size of turbot was estimated to be < 800 Mb [9]. The number of linked SNPs per linkage group varied from 249 in LG 22 to 354 in LG 1, with an average value of 302.14 per LG; the maximum and minimum length of the linkage groups were 157.854 for LG 8 and 70.866 for LG12, respectively (Tables 3 and 4). The estimated genome length was 2639.58 cM (*G*_*e*_). Thus, the genome coverage of this high-resolution genetic linkage map was 99.34% (Table 4).

**Table 2:** Number of candidate SNP markers involved in *S*. *maximus* linkage mapping.

Segregated marker type	Number of linked SNP marker	Average number of missed data
***lm* × *ll***	4667	18
***nn* × *np***	1041	23
***hk* × *hk***	939	18

Where *lm*×*ll* means SNPs are heterozygous in maternity (lm) and homozygous in paternity (*ll*); *nn*×*np* means SNPs are homozygous in maternity (nn) and heterozygous in paternity (np); These two types of SNPs are used for maternal and paternal linkage mapping respectively, and SNPs that are heterozygous in both maternity and paternity (*hk*) are labelled as “bridge” for the final consensus linkage mapping.

**Table 3:** Number of SNP in each linkage group and linkage group lengths (observed and estimated).

Turbot Linkage group	Number of SNPs on linkage group	Linkage group length (cM)	Estimated linkage group length (cM)
**LG1**	354	147.686	148.523
**LG2**	315	96.144	96.756
**LG3**	331	101.789	102.406
**LG4**	327	107.142	107.799
**LG5**	292	137.881	138.829
**LG6**	294	128.768	129.647
**LG7**	278	136.539	137.525
**LG8**	300	157.854	158.910
**LG9**	299	121.393	122.208
**LG10**	298	105.147	105.855
**LG11**	295	153.940	154.987
**LG12**	293	70.866	71.351
**LG13**	335	144.183	145.046
**LG14**	323	134.579	135.415
**LG15**	318	105.340	106.005
**LG16**	316	72.869	73.332
**LG17**	315	109.346	110.042
**LG18**	287	129.095	129.998
**LG19**	281	142.102	143.117
**LG20**	277	108.050	108.833
**LG21**	270	132.810	133.798
**LG22**	249	78.567	79.201
**Total**	6647	2622.090	2639.583

**Table 4:** Summary of turbot (*S*. *maximus*) consensus linkage map.

**Category**	
**Number of markers mapped**	6647
**Average markers number per linkage group**	302.14
**Minimum length of linkage group (cM)**	70.866
**Maximum length of linkage group (cM)**	157.854
**Minimum markers number per linkage group**	249
**Maximum markers number per linkage group**	354
**Average marker interval (cM)**	0.3958
**Observed genome length (*G***_***oa***_ **)**	2622.09
**Estimated genome length (*G***_***e***_ **)**	2639.58
**Genome coverage (%)**	99.34%

### QTL mapping

**Fig 2:**
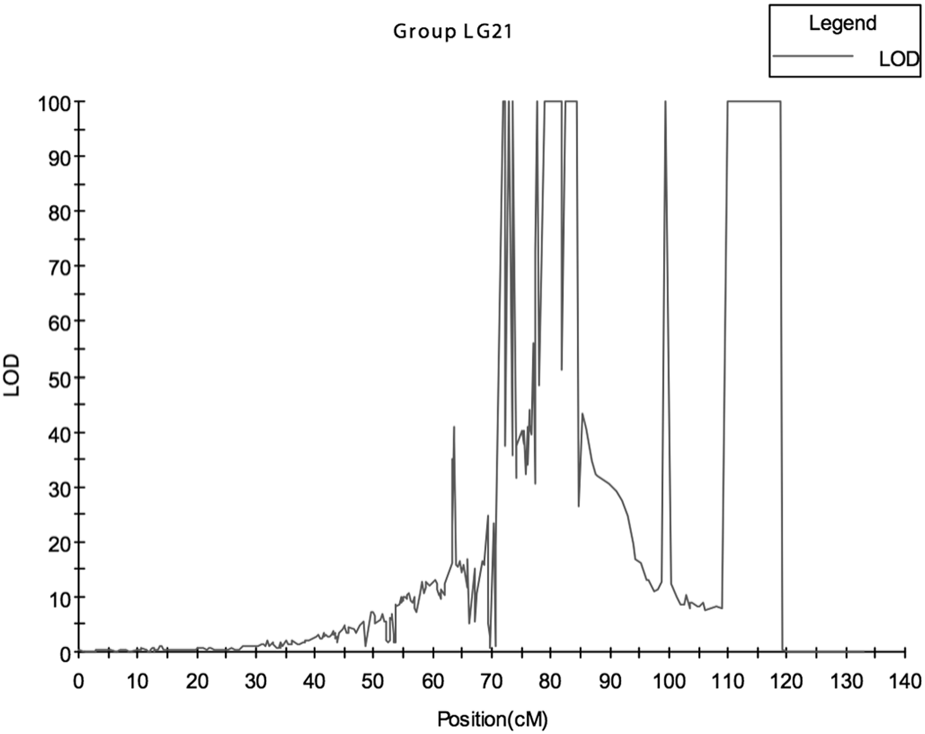
Sex determination QTL mapping in turbot (*S*.*maximus*) LG21. The X-axis represents linkage group, the Y-axis represents LOD scale, and the curved line represents LOD value changes along LG 21. Several QTL regions spanned from 79.08 to 81.839 cM, from 82.424 to 84.267cM, and from 109.905 to 119.005 cM, all displayed the highest LOD scale 99.99, and they formed a “ceiling” LOD region along LG21.

During the entire growth period, the body length (L1–L4) and body weight (W1–W4) of each mapping individual were measured at four different growth periods, and the body thickness (T) of each individual was recorded at the last measurement. Sex was determined by examining the gonad. Briefly, 10 batches of phenotypic data from four phenotypes were involved in the final QTL mapping. The 149 mapping filial individuals were composed of 64 females and 85 males (♀:♂ = 1:1.33), and their growth traits data are listed in Table 5. In total 220, QTL loci corresponding to the five phenotypes (L2, L3, W1, W2, and SEX) with an LOD > 5.0 were identified (the LOD values ranged from 5.86 to 99.99). Of these, 175 linked SNP loci were declared with significant QTL effects were identified, and the remaining 45 QTL summits were located between contiguous SNPs. The number of QTLs corresponding to the five phenotypes (L2, L3, W1, W2 and SEX), were 11, 21, 10, 3 and 175, respectively. The QTL regions were present on 12 LGs, and LG 21 contained the most QTL loci (167). For all of the LGs containing QTLs (QTL-LG), the most types of QTLs detected was three; QTLs associated with W1, W2, and SEX were all detected in LG14, but in most cases, only one type of QTL was detected in an LG. The number of LGs present on a phenotypic QTL ranged from 1–6; the number of LGs containing QTLs related to phenotype L2 was 8, and the number of LGs containing QTLs related to phenotype L3 was only 1. Moreover, the LG for L3 was different from the previous eight QTL-LGs. Unexpectedly, the QTLs associated with sex determination were detected at LG 7, LG 14 and LG 21; however, most were detected at LG 21 (Fig. 2). The different QTL summits, including those that overlapped with SNP loci and those located between SNPs, explain the corresponding phenotypic variance from 14.4–100.0%, with an LOD score ranging from 5.0 to 99.99 (Table 6). All QTLs and SNPs loci were single-function-explained (i.e., they only correspond to one of the five phenotypes), except for SM_343, which was located in LG1 and was associated with both phenotypes W1 and W2.

**Table 5:** Details of growth traits during the entire culture period.

Phenotype	Minimum	Maximum	Average
**L1(cm)**	8.4	10.8	9.29 ± 0.51
**W1(g)**	28	59.9	37.57 ± 6.16
**L2(cm)**	9.5	13.3	11.15 ± 0.65
**W2(g)**	45.43	99.67	66.40 ± 11.02
**L3(cm)**	12.2	16.3	13.84 ± 0.72
**W3(g)**	87.6	192.7	132.92 ± 21.50
**L4(cm)**	13.4	20.6	17.06 ± 1.12
**W4(g)**	108.9	371.6	226.32 ± 45.39
**T(cm)**	1.4	3.4	2.32 ± 0.37

**Table 6:** Summary of QTLs detected in high-density turbot (*S*. *maximus*) linkage map.

Total number of QTL detected				220		
Number of QTLs overlapped with SNP loci				175		
Number of QTLs located between SNPs				44		
Number of LG on which QTL were detected (QTL-LG)				12		
Number of LG on which QTL was not detected				10		
LG on which the most QTLs were detected (QTLs number)				LG 21 (167)		
Phenotypes involved in QTL mapping	Number of QTLs overlapped with linked SNPs	Number of QTLs located between two linked SNPs	Subtotal	Phenotypic variance explained (%)(*R* ^*2*^)	Number of QTL-LG	The highest LOD value a QTL declared with
**L2**	9	2	11	21.6–28.3	8 (LG4, 6, 10, 11, 17, 20, 21, 22)	6.95
**L3**	20	1	21	14.4–17.5	1 (LG 18)	5.86
**W1**	4	6	10	27.8–57.8	3 (LG 1, 4, 14)	7.25
**W2**	3	0	3	25.0–30.8	2 (LG 1, 14)	6.12
**SEX**	139	36	175	15.0–100.0	3 (LG 7, 14, 21)	99.99
**Total**	175	45	220	/	/	/

## Discussion

### *S*. *maximum* high-density linkage map

The first *S*. *maximum* linkage map was traced in 2007, and comprised 242 SSRs in 26 linkage groups [9]. Previously, we reported a medium size linkage map using SSR markers for *S*. *maximum* containing 118 and 125 mapped SSR loci in male and female linkage maps (part of the SSR was shared by the male and female linkage maps), respectively [14]. However, the finite number of mapped markers and sparse inter-marker spaces (6.5 ± 0.5 cM of Bouza’s and an average of 12.9 cM of Ruan’s) made both of these maps difficult to use for effective QTL mapping and evolutionary studies [9, 14]. In 2012, Bouza reported another consensus linkage map based on 438 markers that were distributed across 24 linkage groups in turbot [8], 180 of which were EST-linked. This gene-enriched high-resolution” map was very helpful for QTL mapping and future genome assembling; however, all of the linkage group numbers could not be merged into the final 22 linkage groups. In this study, we constructed a much higher resolution consensus genetic linkage map with SNPs than has ever been reported using the NGS RAD technique. Subject to the finite number of mapped markers, the total linkage map length of the three previous turbot maps ranged from 721.7 to 1402.7 cM [8, 14]. Here, we constructed a 2622.09 cM genome-wide linkage map, which represents 99.34% genomic coverage. According to the estimated turbot genome size (600–800 Mb) and the average marker interval (0.3958 cM), the present map contains a marker approximately every 0.1203 Mb, making it a perfect platform for QTL identification (this claim is verified in the latter QTL mapping portion) and future physical mapping in turbot. Although similar high-density genetic linkage maps based on NGS have been constructed in other teleost fishes, including Atlantic salmon (*Salmo salar*) and Atlantic halibut (*Hippoglossus hippoglossus*) [15, 28], this is the first turbot linkage map with an interval-marker space below 0.5 cM and a physical map distance of 0.2 Mb. Additionally, the number of linkage groups (22) in the present study is consistent with the turbot karyotype haploid 2n = 44 [29], this indicated that each linkage group in the present study corresponds to a turbot chromosome, although other techniques, such as fluorescent *in situ* hybridization (FISH), are needed to anchor the markers to the physical map. Recombination frequency (RF) between the sexes in turbot has been detected in previous studies [8, 9, 14], all of which revealed a higher RF in females than in males (1.3:1 and 1.6:1, respectively; the molecular markers were nearly all composed of SSRs). We did not calculate the RF between females and males. However, the number of segregated and linked markers in the maternity and paternity linkage maps was 4, 667 and 1, 041, respectively, indicating that the SNP RF is also higher in female than in male turbot. Moreover, the RF between male and female SNP markers is higher compared with that of SSRs (Table 2). However, we noted an interesting phenomenon regarding the sex ratio both within the population and the family; the number of females was always lower than the number of males [30–32]. A previous study consisting of 4, 022 four month-old individuals also detected a sex ratio of 1:1.52 between females and males in a farming stock (Unpublished). The female and male ratio was 1:1.33 in this mapping family. Further investigation is needed to determine whether or not a correlation exists between the higher RF in females and lower sex ratio in turbot.

### QTLs correlated with growth mapping

The females turbot exhibited a two-fold faster growth speed than males after 8 months. Thus, clarifications of the genetic mechanisms of growth traits and sex differentiation have been topics of great concern in both turbot farming and academic fields [33, 34]. Among the phenotypic traits of all 10 batches, no QTLs associated with phenotypes L1, L4, W3, W4, and T were detected with an LOD > 5.0 threshold. For body length (L2), 11 QTLs were detected on LGs 4, 6, 10, 11, 17, 20, 21, and 22, including nine that overlapped with linked SNP markers and two located in the region between SNPs. Thus, QTL-L2 was the most widely distributed QTL (Table 6). There was no association between body length (L3) and QTL-L2, as all of the 21 QTL-L3s were detected on LG 18, as opposed to the eight LGs containing QTL-L2. With the exception of one QTL locus, the remaining 20 QTL-L3s all overlapped with linked SNP loci on LG 18 (Table 6). These data suggest that turbot body length is controlled by multiple QTLs, most of which can be traced directly to SNPs and the effect of which varied on LGs and SNPs during different growth periods.

In contrast to the unstable expression of body length QTLs, those related to body weight in two different growth periods (QTLs-W1 and QTLs-W2) were detected on LG1 and LG14, and one related to W1 was also detected on LG4. Moreover, a SNP locus SM_343 on LG1 displayed a significant QTL effect during the first and second growth periods, and this was the only SNP locus (QTL) responsible for two or more traits among all of the 220 with an LOD > 5.0; the remaining 219 QTLs (SNPs) all corresponded to a single trait. A significantly positive correlation was observed between turbot body length and weight in different growth periods [11], which was not supported by the body length and weight QTLs (LOD > 5.0). However, further analysis covering all of the body length and weight QTLs with an LOD > 3.0, a default threshold value to declare a QTL, some interesting results emerged: the number of body length and weight QTLs in all four different growth periods increased from 45 (LOD > 5.0) to 406 (LOD > 3.0). According to their distribution in different growth periods, these 406 QTLs (LOD > 3.0) could be classified into four types: Type I single QTL (SNP) corresponding to any single trait in a specific growth period (n = 243); Type II single QTL (SNP) corresponding to both body length and weight, whether these two traits were in the same or different growth periods (n = 130); in this case, a single QTL was significant in at most three growth periods; Type III single QTL (SNP) corresponding to body length in more than one growth periods (n = 1); Type IV single QTL (SNP) corresponding to body weight in more than one growth period (n = 32), in this case, only one QTL locus was effective in all growth periods (also SM_343 in LG1) (Table 7). The significantly positive correlation between body length and weight gained strong support from 130 Type II QTLs detected in the present study. Regarding the application value of the 44 (LOD > 5.0) (including one SNP related to both W1 and W2) growth trait SNPs (QTLs) for future use in the MAS program for turbot farming, the number and reliability of the QLTs (SNPs), percentage of phenotypic variance explained (*R* ^*2*^) by the markers, distance (cM) of the closest marker to the QTL, and other data should all be considered. Compared with previous growth-QTL studies in turbot [11, 14], not only did the number and reliability (detected QTLs with an LOD > 5.0) of QTLs increase significantly, but most of them (36 out of 44) were also SNPs. Additionally, the growth-QTLs *R* ^*2*^ values (ranging from 14.4–57.8%) were higher than those in previous studies; the highest *R* ^*2*^ was 25.8%, which was detected in a QTL associated with Fulton’s factor in turbot [11]. For the first time in turbot, this study carried out a growth-related QTL analysis with four sets (L1- L4; W1- W4) using continuous data records (Table 6). Moreover, the linkage map density of this QTL analysis is the most saturated ever, providing the opportunity to detect more QTLs. Additionally, there is a greater chance that the QTLs detected would overlap with SNP markers because of the interval region between SNPs, which will increase the accuracy of MAS in turbot. Thus, the newly identified growth-associated QTLs (SNPs) in the present study represent a promising platform for the forthcoming MAS in turbot farming.

**Table 7:** Part of QTLs (LOD > 3.0) related to body length and body weight distribution at four growth periods in turbot (*S*. *maximus*).

		LOD value								
LG No.	SNP(Genetic distance)	L1(*R* ^*2*^)	L2(*R* ^*2*^)	L3(*R* ^*2*^)	L4(*R* ^*2*^)	W1(*R* ^*2*^)	W2(*R* ^*2*^)	W3(*R* ^*2*^)	W4(*R* ^*2*^)	Type
**LG4**	SM_1096(35.323cM)	/	5.26(23.50%)	/	/	/	/	/	/	I
**LG4**	SM_1245(65.646cM)	/	/	/	3.02(10.9%)	/	/	/	/	I
**LG6**	*(62.716cM)	/	4.20(22.3%)	/	/	/	/	/	/	I
**LG6**	SM_1830(89.598cM)	/	/	/	/	/	/	/	3.11(12.9%)	I
**LG1**	SM_343(138.838cM)	4.28(21.9%)	3.21(16.8%)	3.35(20.3%)	/	5.76(27.8%)	6.12(30.8%)	3.72(23.1%)	3.12(10.8%)	II
**LG1**	* (144.268cM)	3.83(35.2%)	/	/	/	7.25(47.6%)	3.38(35.7%)	/	/	II
**LG2**	SM_422(13.316cM)	/	/	3.04(11.2%)	/	/	3.45(13.1%)	3.80(13.6%)	3.20(11.2%)	II
**LG8**	*(124.347cM)	/	/	3.08(12.6%)	3.03(10.8%)	/	/	/	/	III
**LG6**	SM_1811(85.673cM)	/	/	/	/	3.34(27.3%)	4.08(32.2%)	/	/	IV
**LG21**	*(91.955cM)	/	/	/	/	/	/	3.84(13.4%)	3.57(12.7%)	IV
**LG21**	SM_6370(94.336cM)	/	/	/	/	/	/	3.90(13.7%)	3.74(13.6%)	IV

* represents the QTLs are located between two contiguous linked SNPs; / means the LOD score was <3.0; L1–L4 and W1–W4 represent traits of body length and body weight at four growth periods, *R* ^*2*^ represents phenotypic variance explanation (%).

### QTLs correlated with sex determination

Although sexual dimorphism is not so obvious in turbot compared to the half-smooth tongue sole (*Cynoglossus semilaevis*) [35], turbot females exhibit a growth advantage over males. After approximately 8 months, the average weight ratio between females and males begins to grow, reaching 1.8 kg: 1 kg at 20 months [34]. This finding is not only important to the framing industry, where developing all-female stocks is profitable but also to the academic field, where the clarification of sex determination mechanisms, sex-associated QTL detection and related research are points of focus. Despite the fact that a heteromorphic sexual chromosome [29, 36] and sex chromosomal system (ZW/ZZ or XX/XY) have not been determined in turbot [31, 32, 37, 38], many previous studies have reported that the detection of sex-related QTLs or molecular markers (RAPD, SSR) were helpful in clarifying turbot sex determination mechanisms [10, 39, 40]. In this study, which included many more QTLs (SNPs) with high *R* ^*2*^ values, the single QTL and SNP sexual phenotypic variance percentage ranged from 15–100%, and the highest LOD score was 99.99 (Table 6). In contrast to previous expectations that the sex-QTLs would only be detected in one turbot LG, three LGs, LG 7, LG14, and LG21, all possessed either sex- QTLs or SNPs (Table 6). However, number of QTLs (SNPs) and distribution intensity of these LGs were very different: in LG7, a 5 cM sex-QTL region (no linked SNP distributed on it) spanned from 93.799–98.799 cM, and the QTL effect was assessed every 1 cM in this region. An interval QTL model (IM) suggested that all six sites displayed high *R* ^*2*^ values (100%) and high LOD scores (99.99). The closest up-stream SNP marker to this sex-QTL region is SM_2147, which lies at 89.799 cM with a 4 cM space. The closest down-stream SNP marker is SM_2148, which lies at 106.386 cm with a 7.577 cM space. In LG 14, the sex-QTL region covered 3.504 cM, spanning from the 128.381 cM (SNP SM_4331) to the 131.885 cM site. However, SM_4332, which lies at 129.885 cM, did not display a significant QTL- SEX effect. The closest down-stream SNP locus was SM_4333, with 0.339 cM space to the 131.885 cM site. All four sex-QTL had an *R* ^*2*^ value of 100% and an LOD score of 99.99. If only considering the scale of the sex-QTL regions and the number of SNPs detected, LG21 might play a dominant role in sex determination in turbot; the sex—QTL region detected spans 70.882 cM, from 48.183 cM (SM_6246) to 119.005 cM (no overlapping SNPs on this site). The closest down-stream SNP is SM_6395 at 119.342 cM with a 0.337cM space. This 70.882 cM region is almost integrated, but there are still five “empty mosaic” regions, the total length was 3.834cM, where no significant (LOD > 5.0) sex-QTL was detected. Almost all of the linked SNPs (138 out of 149) in this large sex-QTL region possessed significant sex-QTL effects, with an *R* ^*2*^ value for sex determination ranging from 15–100% and LOD scores ranging from 5.24–99.99; the remaining 26 sex-QTL regions were detected between two contiguous linked SNPs with an interval > 1.0 cM.

In total, 175 sex-QTL (SNPs) with an LOD > 5.0 were scattered across LG7, LG14 and LG21. Moreover, most sex-QTL were directly located on SNPs (139 out of 175). This suggests that some of the QTLs (SNPs) can be directly used in MAS, and that recombination is no longer a considerable factor, thus increasing accuracy. Interestingly, among the 36 sex-QTL regions identified in all of the three LGs, 21 displayed the highest *R* ^*2*^ value (100%) and LOD score (99.99); however, this number was only 12 for sex-QTL SNPs. A reasonable explanation for this finding is that the genomic DNA region controlling sex determination in turbot lacks the restriction endonuclease (PstI) site. Alternatively it could be attributed to a self-protection mechanism. The results of our study are very different from previous findings regarding sex-related QTLs and molecular marker detection in turbot. Martinez identified LG5 as the main SD region, on which the closest SSR marker was SmaUSC-E30 with 2.6cM space [10]. Recently, several candidate genes related to sex determination and gonad differentiation were identified in other species, including *Amh*, *Dmrta*2 and one RAPD marker, which were reportedly located on LG5, confirming the major role of LG5 in sex determination; although, these three markers were no closer to SD than SmaUSC-E30, *Amh* and *Dmrta*2 are not considered primary sex determination genes in turbot [40]. Casas isolated four sex-associated RADP markers with sexing efficiencies ranging from 77 to 90% based on different turbot stocks; however, this sex-associated polymorphism disappeared after those RAPD markers were converted to SCAR markers [38]. It is generally accepted that RAPD is not an ideal marker because of its high false positive rate and unstable repeatability. In this study, three sex-QTL regions were identified on three LGs, and they spanned 5.000, 3.504, and 70.822 cM, respectively, which is much longer than the previously confirmed turbot SD region in turbot. This study also detected far more SNP markers related to sex determination (139) in turbot than was previously reported Although most of these displayed a high *R* ^*2*^ value with a high LOD score, they must be verified in multiple families and/or in a large population. Considering the large number of candidate sex-related SNPs and the low recombination frequency among them, haploid analysis would be an efficient strategy for those distributed on the same LG, especially LG21. The interaction or epistatic effect of LG7, LG14 and LG21 should also be considered before making a final conclusion regarding turbot sex-determination mechanisms.

In this study, we detected 6, 647 SNPs distributed on 22 LGs, a number consistent with the turbot karyotype of 2n = 44 in turbot. These SNPs were used to construct a consensus linkage map, which represents the highest saturated linkage map so far. The map contains abundant QTLs related to growth traits in different life stage and sex determination with high confidence. These findings represent a promising platform for MAS and Genomic Assistant Selection (GAS) in turbot and will help clarify the sex-determination mechanisms.

Additionally, each linked SNP corresponds to a unique 85 bp RAD sequence, and a comparison of this consensus linkage map, especially RAD-reads on which QTLs were detected, against other species have already revealed some interesting results. For example, a sex-related SNP, SM_6344 (*R* ^*2*^ = 73%, LOD = 40.35), displayed 85–100% similarity (E value from 4e^05^–3e^56^) with ATPase, a conservative gene in many species: *Homo sapiens* (accession number: XM 006719965.1), *Stegastes partitus* (accession number: XM008300855.1), *Neolamprologus brichardi* (accession number: XM006797868.1) and 10 other species. Moreover, high homology between LG21 in *Cynoglossus semilaevis* and LG21 in turbot was also observed (Unpublished data).
